# What Role Does CFTR Play in Development, Differentiation, Regeneration and Cancer?

**DOI:** 10.3390/ijms21093133

**Published:** 2020-04-29

**Authors:** Margarida D. Amaral, Margarida C. Quaresma, Ines Pankonien

**Affiliations:** BioISI—Biosystems and Integrative Sciences Institute, Faculty of Sciences, University of Lisboa, 1749-016 Lisboa, Portugal

**Keywords:** carcinogenesis, cystic fibrosis, epithelial–mesenchymal transition EMT, epithelial differentiation, epithelial regeneration, wound healing

## Abstract

One of the key features associated with the substantial increase in life expectancy for individuals with CF is an elevated predisposition to cancer, firmly established by recent studies involving large cohorts. With the recent advances in cystic fibrosis transmembrane conductance regulator (CFTR) modulator therapies and the increased long-term survival rate of individuals with cystic fibrosis (CF), this is a novel challenge emerging at the forefront of this disease. However, the mechanisms linking dysfunctional CFTR to carcinogenesis have yet to be unravelled. Clues to this challenging open question emerge from key findings in an increasing number of studies showing that CFTR plays a role in fundamental cellular processes such as foetal development, epithelial differentiation/polarization, and regeneration, as well as in epithelial–mesenchymal transition (EMT). Here, we provide state-of-the-art descriptions on the moonlight roles of CFTR in these processes, highlighting how they can contribute to novel therapeutic strategies. However, such roles are still largely unknown, so we need rapid progress in the elucidation of the underlying mechanisms to find the answers and thus tailor the most appropriate therapeutic approaches.

## 1. Introduction

With the recent advances in cystic fibrosis transmembrane conductance regulator (CFTR) modulator therapies and the substantial increase in life expectancy of individuals with cystic fibrosis (CF), novel challenges are emerging at the forefront of this disease. One of the key features associated with this long-term survival rate is an elevated predisposition to cancer. In fact, although already described for a long time, the inherent predisposition of individuals with CF to develop cancer has been firmly established by recent studies involving large cohorts [1,2,3].

**Figure 1:**
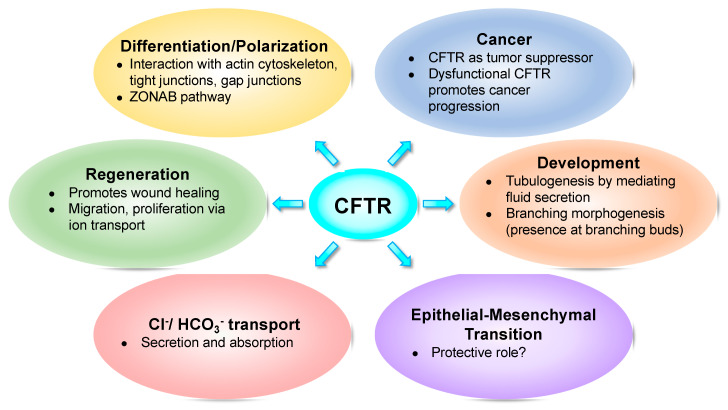
Cystic fibrosis transmembrane conductance regulator (CFTR) impacts several fundamental cellular processes.

In parallel with cancer, an increasing number of emerging studies show that CFTR plays a role in fundamental cellular processes such as foetal development [4,5,6,7,8,9], epithelial differentiation/polarization [10,11,12,13,14], regeneration [15,16,17], and epithelial–mesenchymal transition (EMT) [18,19,20,21,22] (Figure 1).

In parallel, multiple studies also report an actual functional role for CFTR as a tumour suppressor gene, whereas dysfunctional CFTR promotes cancer development both in vitro and in vivo [18,23,24,25,26]. Which mechanisms link dysfunctional CFTR to carcinogenesis remains an open question.

Although many of these studies provide only circumstantial correlations between CF and cancer, it remains unclear whether carcinogenesis is a direct consequence of dysfunctional CFTR, and the compelling accumulated evidence calls for urgent investigation.

Clues to this challenging question derive from findings in foetal development, during which CFTR evidences highly specific spatio-temporal expression patterns as multiple organs develop [27,28,29]. CFTR was proposed to play a key role in processes such as tubulogenesis (e.g., vas deferens) and branching morphogenesis (of airways, pancreatic or kidney ducts). Some light has been shed by suggesting that such roles may essentially derive from CFTR´s major function in regulating fluid secretion and, consequently, in promoting mechanical stretching.

Another noteworthy question is how CFTR is implicated in epithelial differentiation/ polarization and in the related process of tissue repair. Data collected from wound healing experiments, as an experimental model to study these processes, clearly show that functional CFTR promotes migration while also affecting proliferation. Thus, acting on these two processes could be a novel strategy to promote epithelial repair in CF.

Concrete evidence for the role of CFTR in epithelial differentiation also derives from the observation that its absence leads to an intermediate (partial) state of EMT (M Quaresma and Amaral lab, unpublished data). Moreover, EMT induction by dysfunctional CFTR also provides a direct (causal) link to its role as tumour suppressor. However, further studies are needed to characterize how functional CFTR prevents progression into EMT.

In the following sections, we provide state-of-the-art descriptions on the moonlight role(s) of CFTR on these processes, highlighting how the understanding of the underlying mechanisms can contribute to novel therapeutic strategies. However, such roles are still largely unknown, so we need rapid progress in the elucidation of these mechanisms to find the answers and thus tailor the most appropriate therapeutic approaches.

## 2. CFTR and Development

### 2.1. CFTR Expression is Highly Regulated During Development

A rationale for the possible different roles of CFTR during development stems from its finely regulated spatio-temporal expression during foetal development.

CFTR mRNA is present at high levels very early in several organs during human foetal morphogenesis (see Table 1), namely in developing pancreas (ducts and centroacinar cells), liver (bile duct and gallbladder epithelium) and intestine (crypts) in the first trimester (10–12 weeks of gestation, WG). Lower but significant expression levels are also detected in the epithelia of small airways and trachea as well as in the ductal epithelium of the epididymis (largely in the vas deferens) at this stage. The uterus, fallopian tubes and salivary glands also present CFTR expression but only in the third trimester. In most of these tissues (e.g., pancreas, intestine, reproductive system), the pattern of CFTR expression in the mid-trimester human foetus and in the adult are similar. The major exception to this is the lung, where high levels of CFTR are present through mid-trimester in marked contrast to the reduced expression in the adult lung [27]. Measurable levels of CFTR transcripts are present in the primordial epithelium of the pseudoglandular stage lung [28]. In these immature lungs (as early as 7 WG), CFTR is diffusely expressed in the cytoplasm of undifferentiated multipotential stem cells [29].

The highest levels of CFTR mRNA expression occur at the start of the second trimester and gradually decline to term [27,34]. Similarly, CFTR protein expression increases until around 20 WG after which it is gradually down-regulated, starting at 23–24 WG and continuing until birth [35]. This temporal window is consistent with lung maturation and cell differentiation (24–25 WG), where CFTR localization shifts to an apical localization in ciliated cells, also being present in the collecting ducts and glands of the airways, as in the adult lung [29]. This shift from non-polarized to a prominent apical localization of the bronchiolar epithelial cell was also described for CFTR in developing rabbit airways [36].

It has been postulated that the marked developmental changes in both the amount and local distribution of CFTR expression in the foetal and postnatal human lung may be consistent with the changes that occur in the lung transition from a fluid-secreting to an absorbing organ (Na^+^/Cl^−^ bulk flow) close to term [28]. However, since CFTR down-regulation starts mid-term, it does not appear to coincide with the transition from intra-uterine to post-natal life, but is rather specifically connected to lung development/differentiation [35]. It is possible that CFTR has a particular role in the differentiation of the respiratory epithelium, e.g., mutant CFTR protein would fail to regulate important genes during early gestation resulting in abnormal lung fine structure and function [34,35]. However, such role(s) are still unknown.

### 2.2. Individuals with CF Display Lung Malformations and CBAVD

Supporting an important role for CFTR in lung development, early studies on the airways of CF foetuses described morphologically abnormal tight junctions (TJ) often accompanied by ciliary defects [37], including absence of cilia in most tracheal epithelial cells, as well as tracheal epithelial atrophy [38]. Subsequent studies found that mice, rats, pigs and newborns/young children who lack functional CFTR display developmental and functional defects in trachea, such as malformations of the cartilaginous rings, reduced breathing rate, decreased contractile response, decreased lumen size and circularity and increased smooth muscle tissue [6,39,40].

In CF pigs, these morphological lesions are detected early in the pseudoglandular stage of lung development and during early branching morphogenesis of the proximal airways, where most defects are found after birth [41]. Furthermore, rats subjected to transient in utero CFTR knockout displayed with aging, even after re-activation of CFTR expression, airway thickening and fibrosis, a classic CF phenotype [42]. In agreement, transient gene transfer of CFTR into normal foetal mice [43,44] and rats [45] increased both bronchial cell proliferation and differentiation, namely of secretory cell types. In utero gene therapy was also applied to non-human primates showing similar increases in cell proliferation and stimulation of differentiation of primordial stem cells to mature into secretory cells [46].

All these studies point to CFTR being instrumental for the normal differentiation of secretory cell populations in the developing airways. Most importantly, they show that small transient changes in CFTR expression during lung development have substantial effects on organogenesis [4], reinforcing its central role in development.

Another major body of evidence on the role CFTR plays in development is that 97–98% of male individuals with CF are infertile, mostly due to congenital bilateral absence of vas deferens (CBAVD) [47,48,49]. Absence of vas deferens and markedly reduced or partially absent epididymis in CF individuals, with subsequent azoospermia, has been reported since 1968 [50]. Dysfunctional CFTR is proposed to result in disordered morphogenesis of the Wolffian duct derivatives by generating abnormally thick and sticky mucus which clogs the vas deferens as it is forming, causing its degeneration before birth [9,27,30,51]. Interestingly, a CFTR^-/-^ rat model also presented loss of the vas deferens by six weeks of age [40], suggesting that the role of CFTR in development of male reproductive tract may be common across species. However, at least two studies suggest that normal vas deferens are present during development, and its absence happens by progressive atrophy/degeneration due to less functional CFTR and mucus obstruction over time [52,53]. 

CBAVD is itself a condition that occurs in about 1–2% of infertile, but otherwise healthy, men [9]. It is intriguing that such a large percentage of male individuals with CF have CBAVD. In fact, between 1990 and 2000, several studies found that a large proportion of individuals with CBAVD (41–71%) are actually carriers of CFTR mutations, many being compound heterozygotes [51,54,55,56,57]. These findings suggest that the majority, if not all, cases of CBAVD might be due to CFTR mutations [51], a notion that is increasingly accepted. Thus, CBAVD, along with other conditions, can be considered a “CFTR-opathy”, a term coined to designate CFTR-related disorders other than CF [58]. In fact, a meta-analysis of literature reporting CFTR mutations and CBAVD between 1992 and 2011 indicates that 78% of individuals with CBAVD have at least one CFTR gene mutation (53% having two and 25% only one). Furthermore, F508del/5T and F508del/R117H are the two most common compound heterozygote genotypes of men with CBAVD, which clearly differ from those occurring in typical CF [48,59]. Indeed, R117H [55] and the 5T polymorphism [60] are the most frequent CFTR variants found in individuals with CBAVD.

Importantly, the fundamental difference between CF and CBAVD (as for other “CFTR-opathies”) is the distribution of genotypes: while individuals with CF present two severe mutations (~89%) or one severe and one mild mutation (~11%), those with CBAVD have either a severe and mild (~89%) or two mild (~12%) mutations, i.e., those with residual function and included in classes IV, V or VI [57,61,62].

Hence, most individuals with CBAVD are at present considered to have a “CFTR-opathy” (previously also considered to be a “genital” or “incomplete” form of CF). This prompts the question of why the reproductive tract is so sensitive to CFTR mutations compared to other organs. The reason for this is probably two-fold. Firstly, non-CFTR modifier genes and/or environmental factors, both being main contributors to different phenotypes arising from similar genotypes, may impact different organs with different magnitudes [61]. Since CBAVD is present in >95% of CF individuals, most likely (mutated) CFTR is the major contributor to this condition, with other genes/environment playing minor roles. Lung disease, on the other hand, displays a wide range of severity in individuals with CF, suggesting that the environment and/or modifier genes are major contributors to the phenotype in the airways [63]. Secondly, another possibility is differences in alternative splicing of CFTR mRNA in different tissues. Splicing of mRNA has been found to be less efficient in vas deferens than in respiratory epithelia, which implies that the reproductive system is more sensitive to CFTR protein dysfunction than other tissues [9]. For example, a 90% reduction in the number of transcripts resulting from the T5 allele is enough to cause malformations in the vas deferens but not in the lung or pancreas [64]. CFTR expression in nasal epithelium is actually considered to be independent of the Tn genotype [9,60,64]. Nonetheless, this latter argument would only account for differences in splicing mutations, but not other common CBAVD mutations (e.g., R117H, V201M, I980K, D1270N, etc.).

### 2.3. What Is the Major Role of CFTR in Development?

Although to date the exact role(s) of CFTR during embryonic/foetal development remains unknown, the three main possibilities proposed thus far are that CFTR is critical in: (1) pre-implantation embryonic development; (2) correct branching morphogenesis and tubulogenesis; and (3) promoting correct terminal lung differentiation of the distal airways. 

Firstly, CFTR protein expression and activity was found to be expressed in human pre-implantation embryos, as early as the 8-cell stage, but also in morulae and blastocysts [65]. Most studies, however, have been conducted in mice, where CFTR has also been suggested to be essential for the embryonic cleavage phase and for embryo differentiation. It was first proposed that CFTR was directly responsible for regulating HCO_3_^-^ secretion in the oviduct [66] as a soluble adenylate cyclase (sAC)-dependent, cAMP-activated HCO_3_^-^ channel. Absence of HCO_3_^-^ is detrimental beyond the 2-cell embryonic stage, and development of mouse embryos from this stage to the morula or blastocyst failed when co-cultured with HCO_3_^-^ secretion CFTR-deficient epithelial cells [67]. Since then, it has been shown that cleavage-associated events are also dependent on Cl^−^, being mainly mediated by the Cl^−^/HCO_3_^−^ exchangers SLC26A3 and SLC26A6. However, these need active CFTR Cl^-^ channel to provide an essential recycling pathway for this ion. Defects in either the SLCs or CFTR disrupt the HCO_3_^-^-dependent signalling cascade and suppress embryo cleavage [68]. The complete mechanism involves HCO_3_^-^ secretion by the SLCs (supported by CFTR), activating sAC, generating cAMP that activates protein kinase A (PKA), triggering NF-κB, which in turn regulates miR-125b which down-regulates p53, a requirement for early embryo development [68].

The loss of CFTR function (by injection of CFTR antisense morpholino) in 2-cell-stage *Xenopus laevis* embryos also caused ~73% of them to die and the remaining 27% to exhibit various malformations. Accordingly, CFTR morphants presented significantly down-regulated mesoderm and endoderm marker genes, as well as significantly reduced levels of the Wnt/β-catenin (β-cat) downstream targets. The Wnt/β-cat signalling pathway has a central role in controlling proliferation and lineage specification during early embryogenesis. CFTR appears to be critical for early embryonic development by regulating β-cat-associated pathways in vivo [8]. Additional results from mouse embryonic stem cells (mESCs) point to a role of CFTR in early mesendoderm differentiation, by interacting with β-cat and preventing its phosphorylation and premature degradation [8]. The loss of CFTR is proposed to result in suppression of β-cat transcriptional activity affecting Wnt signalling that is instrumental in mesendoderm induction [8]. Interestingly, wt-CFTR itself is known to interact with several components of the Wnt signalling pathway, an association which is lost for F508del-CFTR [69]. However, another study in *Xenopus laevis* embryos suggested that CFTR-mediated retinoic acid signalling through retinoic acid receptor alpha (RARα) is the critical pathway for embryo development [70]. The loss of CFTR was found to impair the migration of primordial germ cells (PGCs, undifferentiated stem cells that are precursors of gametes) in zebrafish, confirming a CFTR role in early embryogenesis [71].

Secondly, CFTR was described as influential in controlling mechanical morphogenesis during development. Mechanical forces are as crucial as genes and chemical signals in the control of embryonic development, morphogenesis and tissue patterning [72]. Two major developmental processes, tubulogenesis and branching morphogenesis are highly dependent on the concerted actions of mechanical forces and genetic programmes. Tubulogenesis, a key process underlying the development and structural organization of glandular organs, consists in the coordinated proliferation, polarization and reorganization of epithelial cells to form a lumen and then in promoting its expansion [73,74]. Branching morphogenesis is the developmental process leading to complex ramified epithelial networks/trees of tubes that support the flow of gases and fluids, occurring in the airways, in the kidney collecting ducts and the ducts of mammary and salivary glands [75,76].

The main driver of cellular mechano-transduction in development was first suggested to be cytoskeletal tension, as its inhibition through the Rho/Rho-associated protein kinase (ROCK) pathway resulted in abnormal mouse lung development with suppression of both epithelial budding morphogenesis and branching angiogenesis [77]. However, another study in embryonic rat lungs suggested that ROCK alone was not sufficient to induce proper growth and differentiation [5]. CFTR was proposed to increase extracellular Cl^-^ concentration, stimulating ATP release, increasing the synthesis of muscle proteins and releasing Ca^2+^ necessary for foetal airway contractions through a PI3K-PLC-CK2 pathway. In the absence of functional CFTR, lung cytoskeleton tensions would be only dependent on ROCK and normal foetal movements, causing growth and differentiation to occur at a slower rate [5]. This could account for the deficiencies in function, which become more apparent as the CF lung ages and is subjected to environmental insults.

However, in further studies of branching morphogenesis and tubulogenesis, it became apparent that developmental mechanical forces are greatly affected by changes in ion secretion and ionic gradients, with fluid flow and hydrostatic pressure being responsible for shaping morphogenesis at several levels [74]. For example, transmural pressure, which is controlled by the relative pressure of the fluid contained within the lumen, has been found to drive airway branching morphogenesis. Higher transmural pressure leads to an increased growth rate of both the epithelium and the mesenchyme, an increase in the complexity of the airways, and to the expression of lung developmental programmes, i.e., enhancing the expression of genes driving airway branching morphogenesis, such as fibroblast growth factors (FGFs) and genes involved in airway maturation such as surfactant proteins and mucins [76]. These studies illustrate how fluid secretion is an important controller of these mechanical signals and importantly, how CFTR, a master regulator of fluid secretion can impact on those processes. CFTR was indeed shown to play a major role in regulating the lumen size of epithelial MDCK cell acini by controlling apical secretion of Cl^-^ ions that generate water influx, thus creating an osmotic pressure within the lumen [22]. CFTR inhibition was also found to prevent the formation of tubular structures in a 3D epididymal cell culture model [78]. CFTR has been reported to be important in lumen expansion during tubulogenesis of the developing submandibular gland. While vasoactive intestinal peptide (VIP) promotes lumen formation and expansion through different mechanisms, a VIP-responsive PKA/CFTR-dependent mechanism is involved in lumen expansion [73]. Accordingly, in zebrafish, the loss of CFTR-mediated fluid secretion severely impairs Kupffer’s vesicle lumen expansion [7,79] leading to defects in organ laterality, i.e., left-right asymmetry [7]. Regulation of CFTR activity-mediated fluid secretion was also shown to be critical during zebrafish gut development [80]. It is thus safe to propose that CFTR plays a role in tubulogenesis by mediating fluid secretion.

In parallel, CFTR also appears to play a role in branching morphogenesis. At the pseudoglandular stage of developing pig airways, CFTR protein was exclusively localized to the leading edges of budding airways in non-CF (but not CF) lungs [41]. At 10 WG of human lungs normal CFTR was also seen in the epithelial structures of the pulmonary buds [35]. Interestingly, at this stage no CFTR was detected in the lungs of individuals with CF, suggesting that the defects seen in lung can be traced back to faulty CFTR expression in early lung budding. In fact, human CF foetuses (10 F508del-homozygous; 3 F508-heterozygous) revealed a three-week delay in CFTR protein detectability in the lung, with the protein appearing only at 15 WG [35]. Additionally, CFTR´s direct effect on stretching was also proposed to play a role in late lung development of the distal airways by its levels directly correlating with those of the parathyroid hormone-related protein (PTHrp) and inversely with the Wnt signalling pathway, the latter required for terminal differentiation of the alveoli [81]. While in early lung development, CFTR would promote mechanical stretching for proximal airway development through an initial increase in its expression levels, at later stages a drop in its levels, by as much as 75-fold [34], would instead promote a decrease in pressure and allow Wnt signalling activation for alveolar differentiation.

Strikingly, a change in CFTR expression during human pancreatic development from the termini of pancreatic ducts (areas of developing acini) to the distal portion after birth, suggests that CFTR is also important for branching morphogenesis of the pancreatic acini [32]. CFTR expression was also found in human developing kidney, as early as 12 WG, predominantly in the PM region of the epithelial cells lining the branching ureteric bud [33]. Accordingly, the loss of CFTR during zebrafish development leads to the destruction of pancreatic acinar tissue, suggesting an important function of CFTR in the developing pancreatic ducts of larval zebrafish [82]. Thus, CFTR´s roles in tubulogenesis and branching morphogenesis may be conserved across different organs or even species.

Conceivably, the loss of CFTR during human development does not lead to extreme developmental defects, but the resulting reduction in internal fluid pressure causes the reported foetal development abnormalities. This can happen, for example, by influencing downstream signalling pathways that are important in development, or simply by insufficient expansion of important structures, like lumina. It has long been known that alterations of normal fluid dynamics produce developmental lung anomalies [83]. Whatever the mechanism(s) by which CFTR regulates embryonic development there is no doubt that it plays a relevant role in this process. 

## 3. CFTR and Epithelial Differentiation

### 3.1. Is CFTR Essential for Epithelial Differentiation?

The first evidence for the possible involvement of CFTR in epithelial cell differentiation came from the human intestinal cell lines Caco-2 and HT-29 [84,85,86,87], as CFTR mRNA levels were found to increase as cells differentiated/increased confluency. In nasal polyp tissue (upper airways), wt-CFTR was found to be localized at the apical membrane of ciliated cells in areas of differentiated epithelium, while it localized to the cytosol of poorly differentiated areas of the epithelium, similarly to F508del-CFTR in most cell types [10,11]. Apical expression of CFTR was confirmed in parallel with cytokeratins (CK) 13, 18, 14, or desmoplakin (DP) 1, all markers of full epithelial differentiation. Hence, CFTR expression level appears to be tightly associated with the degree of epithelial differentiation [11,12,88]. This is supported by further studies revealing that wt-CFTR is intracellularly expressed in ciliated cells in a remodelled airway surface epithelium from non-CF individuals, thus demonstrating that airway remodelling and inflammation play critical roles in the differentiation state of the surface epithelium and consequently on CFTR apical localization in both CF and non-CF airways [11,12].

**Figure 2:**
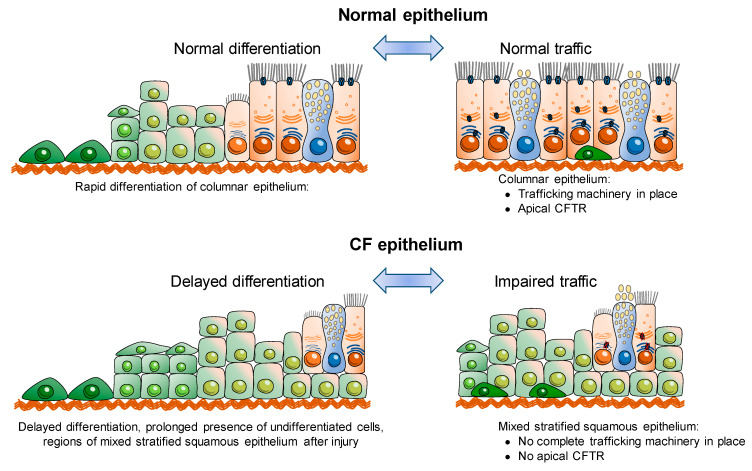
Interplay of CFTR, epithelial differentiation and secretory traffic.

Interestingly, later reports did find that F508del-CFTR also localizes to the apical membrane of some terminally differentiated cells, albeit at significantly lower proportions [89,90]. Altogether, these studies indicate that abnormal CFTR localization, and thus trafficking, is not only due to defective folding of mutant CFTR, but also dependent on the differentiation state of the cell (Figure 2) [11,12,89,90,91].

Furthermore, it was also reported that differentiation of kidney epithelial cells grown on permeable supports leads to an increased plasma membrane (PM) expression of F508del-CFTR. The authors thus propose that an induced polarization/differentiation of the cells leads to increased F508del-CFTR traffic from the ER to the trans-Golgi network and PM [92]. These studies further confirm that the differentiation state of the cell influences secretory traffic of CFTR (Figure 2) and likely also of other PM proteins. The identification of a PDZ-binding domain at the C-terminus of CFTR protein provided a link between polarization state of a cell and CFTR traffic to/stability at the apical PM [13] (see also next section). In fact, a study reported that F508del-CFTR can be rescued to the cell surface without applying any correctors and solely by promoting the anchoring of this mutant to the actin cytoskeleton and PM via its PDZ-binding domain by stimulation of the Rac1 signalling pathway [93] (also see next section).

On the other hand, evidence that CFTR itself plays a role in epithelial differentiation came from human nasal epithelial cells cultured and differentiated on permeable supports. Increased CFTR mRNA expression was found together with an increased expression of mucins and aquaporins suggesting this could induce cell differentiation into a pseudostratified epithelium [94]. Furthermore, genes related to cilia biogenesis were identified to be down-regulated in CF vs. non-CF human native nasal epithelial transcriptomes [95]. Altogether, these observations point to the fact that CFTR expression and/or function is tightly linked to epithelial differentiation/polarization. 

### 3.2. CFTR, Actin Cytoskeleton, Tight Junctions and Differentiation

Several emerging studies have started to shed some light on how CFTR affects epithelial differentiation and polarization and whether its ion channel activity or another one is relevant for this role. CFTR´s association with the actin cytoskeleton, tight and gap junctions (TJs, GJs) seems to be the link between CFTR and epithelial differentiation/ polarization. The polarity of epithelial cells, leading to the selective distribution of membrane proteins between apical–basal sides, is mainly maintained and regulated intracellularly by the actin cytoskeleton [96]. At the membrane itself, separation is provided by the cell junctions—TJs, GJs and adherens junctions (AJs)—that are directly linked to the actin filaments [97,98]. Besides reports on the need for a well-organized actin cytoskeleton for CFTR activation and regulation [99,100], a direct interaction of CFTR with actin was also reported [101]. Furthermore, the KRT8/KRT18 heterodimeric intermediary filaments of the cytoskeleton which are a feature/phenotype of epithelial differentiation were also shown to be an essential component for correct CFTR targeting to the PM [102]. Indeed, several authors found that human bronchial epithelial cells expressing F508del-CFTR show a lack of stress fibres resulting in a disorganized actin cytoskeleton in comparison to wt-CFTR expressing cells [103,104,105]. Similarly, Monterisi et al. found that knocking-down wt-CFTR in human bronchial epithelial cells also leads to a loss of cytoskeleton organization [106] thus suggesting an important role of CFTR in the formation/ maintenance of the cytoskeletal organisation of epithelial cells. Another protein that links CFTR via its PDZ-binding domain to the cytoskeleton is the scaffolding protein Na^+^/H^+^ exchanger regulatory factor isoform 1 (NHERF1) through its interaction with ezrin, which is a PKA anchoring protein connected to the actin cytoskeleton [107,108,109]. It was shown that NHERF1 overexpression increased F-actin redistribution at the apical membrane through its interaction with phosphorylated ezrin which further lead to the rescue of F508del-CFTR from the cytoplasm to the PM in human bronchial epithelial cells [103,110]. Furthermore, stimulation of endogenous small GTPase Rac1 signalling (via hepatocyte growth factor, HGF) was shown to retain F508del-CFTR at the PM of human bronchial primary cells by promoting its NHERF1-mediated anchoring to the actin cytoskeleton [93]. Thus, NHERF1 positively regulates actin cytoskeleton organization and thereby stabilizes CFTR at the apical membrane.

Regarding the interaction of CFTR with TJs, it was observed that the transepithelial electrical resistance (TEER), which is a measure of epithelial tightness [111], is higher in wt-CFTR compared to F508del-CFTR expressing cells when cultured under air-liquid interface (ALI) conditions [14,112,113]. In line with these findings, Castellani et al. observed an accurate organization of zona occludens protein 1 (ZO-1) in non-CF human bronchial epithelial cells, while CF human bronchial epithelial cells (F508del/F508del), besides lower TEER, also showed a disorganized ZO-1 pattern [110]. Inhibiting CFTR function using CFTR_Inh-172_ or low Cl^-^ medium did not alter the TEER suggesting that epithelial tightness/TJ assembly (and thus epithelial differentiation) is not regulated by CFTR anion channel activity [14]. A recent study confirmed the more diffused ZO-1 expression in CF vs. non-CF human bronchial epithelial cells (M Quaresma and Amaral lab, unpublished data). Interestingly, Ruan et al. identified the pathway activated by transcription factor ZO-1-associated nucleic acid binding protein (ZONAB) as a possible mechanism for the regulation of TJs and epithelial differentiation by CFTR. This study shows that CFTR interacts through its C-terminal PDZ domain with ZO-1 and thus colocalizes with ZO-1 at the TJs of trachea and epididymis [78]. In the proposed model, CFTR keeps ZONAB in TJs through its interaction with ZO-1, thus activating epithelial differentiation and reducing proliferation. When mutated, CFTR is retained in the ER, ZONAB translocates to the nucleus thus leading to increased proliferation and decreased differentiation [114]. Although plausible, this hypothesis does not account for the differentiation impairment also observed in CF tissues with other (non-traffic) mutants (M Quaresma and Amaral lab, unpublished data). Early studies, showing that C-terminal PDZ domain of CFTR is essential for its PM localization/stability (see previous section), already suggested that this possibly happens through CFTR binding to ZO-1 [13]. Furthermore, some members of the claudin protein family, which are known to interact with ZO-1, and thereby contributing to the TJ organisation [115], were shown to be differentially expressed in CF vs. non-CF human bronchial epithelial cells: claudin-1 and 4 being expressed in non-CF cells and only claudin-1 being expressed in CF cells, while claudin-4 expression was lost during polarization [110]. Furthermore, decreased expression levels of claudin-1, 7, and 8 and increased expression of claudin-2 were found in CF intestine compared to control [116]. In parallel, GJ proteins were also found to be affected in CF [113,117]. Due to defective trafficking of F508del-CFTR in human bronchial epithelial cells, the GJ protein connexin-43 (CNX43) was found to be mislocalized to perinuclear regions compared to non-CF cells where it showed correct appearance at GJs by punctate staining along cell borders [113]. In a recent study, a disorganized pattern of CNX43 as well as of AJ protein β-cat was also found, both showing a more diffused distribution in CF human bronchial epithelial cells in comparison to non-CF cells (M Quaresma and Amaral lab, unpublished data).

Taken together, these data emphasize a tight connection between CFTR, the actin cytoskeleton, TJs and GJs and that dysfunctional CFTR most probably causes a loss of cytoskeletal organisation and intracellular junctions. However, further studies are needed to unravel the precise role of CFTR in regulating this complex network. 

## 4. CFTR and Regeneration 

### 4.1. CFTR is Needed for Proper Epithelial Regeneration

**Figure 3:**
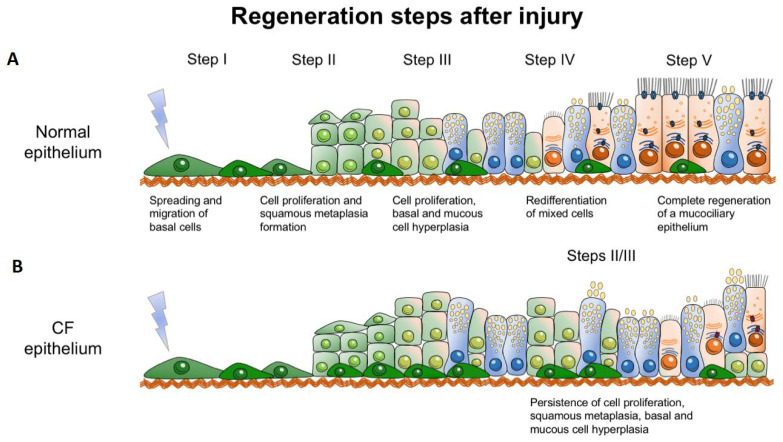
Steps of epithelial regeneration in normal (**A**) and CF (**B**) epithelia (see text for details). [Adapted from: [15,130]].

The repair of epithelial tissue is essential to restore the integrity of the epithelial barrier function upon infection, inflammation, or injury. Epithelial regeneration includes the steps of cell adhesion and migration, where cells cover the wounded area, before cells start proliferating to fully form a new tight monolayer. Thereafter, epithelial cells start to re-differentiate until they are completely differentiated, thus reconstituting a polarized and intact epithelium. [118,119,120]. CFTR was first implicated in tissue regeneration by studies in rat livers. The authors found that, after partial hepatectomy, CFTR mRNA expression levels were upregulated during the regenerative process of the liver [121]. Castillon et al. used a 3D-culture (spheroids) of human airway epithelial cells, previously developed by Jorissen et al., to study the regeneration of nasal epithelium [122,123]. After dissociation (de-differentiation) of human airway epithelial cells, the loss of ciliated cells with concomitant disappearance of the CFTR PM expression was observed. The isolated cells formed spheroids within 24h and re-differentiation was observed after one month in parallel with CFTR expression at the apical PM as well as ZO-1, ezrin, and CD59 (glycophosphatidyl-inositol-anchored protein) [122,124]. Hajj et al. created a humanized airway xenograph mouse model to compare the regeneration process in human non-infected CF and non-CF nasal epithelia [16]. Epithelial regeneration, which includes the steps of cell adhesion, migration, proliferation, pseudostratification and differentiation (Figure 3A), was seen in both CF and non-CF regenerating tissues. However, the CF epithelium showed a delayed differentiation vs. the non-CF tissue, which was associated with an increased proliferation during regeneration, even in the absence of inflammation (Figure 3B, [15,16]). Importantly, several other studies also reported an increased proliferation in CF [95,125,126,127]. In a subsequent study, Adam et al. confirmed a delay in ciliated cell differentiation in nasal airway CF cultures in the absence of secondary symptoms, e.g., infection or inflammation [128]. The fact that impaired regeneration in CF airway cells could be partly improved by applying CFTR modulators is suggestive that functional CFTR, and thus its ion transport, is essential for airway epithelial repair [129].

Furthermore, one study found that genes associated with de-differentiation and epithelial injury are strongly associated with the CF gene expression profile in human native airways [19]. However, it is not yet clear whether the altered regeneration in CF is only due to defective CFTR or the result of chronic inflammation caused by defective CFTR, or both. Interestingly, there is some evidence showing that senescent markers are upregulated in CF, thus suggesting that senescent cells which are proinflammatory contribute to the chronic inflammation in CF [131].

A recent study comparing fully differentiated CF and non-CF human primary airway epithelial cells undergoing repair in absence and presence of flagellin to mimic *Pseudomonas* infection identified differences in the expression levels and times of marker genes distinctive of the various cell types [132]. The mechanisms regulating airway epithelial cell differentiation and regeneration in human tissues are not well understood, thus, this study sheds new light on those processes and the respective genes involved [132]. However, these still require further investigation to understand their mechanistic role in repairing epithelia and their relationship to CFTR.

### 4.2. CFTR and Wound Healing as a Model of Physiological Regeneration

Wound healing experiments on airway epithelial cell models are widely used to study the physiological role of CFTR in epithelial repair of native tissues. Such experiments are performed on cell monolayers either grown on cell plates (non-polarized) or on filter supports (polarized) where a wound is mechanically induced, and the closure of the wound gap is monitored [133]. This process of wound closure usually involves both migration and proliferation of the cells. Using non-CF and CF human airway epithelial cell lines, Maillé et al. observed a significant delay in cell migration for CF relative to non-CF cells. However, no significant effect was seen when non-CF cells were treated with CFTR inhibitor CFTR_Inh_-172 [134]. The authors related the delay rather to defective EGFR signalling and reduced potassium channel function [134]. In contrast, several other studies relate the delay in wound healing to CFTR dysfunction. Schiller et al. used siRNA and CFTR_Inh_-172 to silence CFTR expression or function in the Calu-3 cell line and in primary human non-CF bronchial epithelial cells. This study shows that both downregulation of CFTR expression and inhibition of CFTR function impaired wound closure, thus indicating that CFTR-mediated ion transport plays a role in cell migration/proliferation. Interestingly, they further observed reduced lamellipodia protrusion when silencing CFTR, thus, suggesting that the role of CFTR is exerted, at least partially, at the level of cell migration [17,135]. Trinh and colleagues performed wound healing experiments using nasal and bronchial airway cells from individuals with CF and controls and observed a delay in wound closure for the CF cells [136]. Furthermore, downregulation of wt-CFTR expression inhibited wound closure and treatment with CFTR inhibitor GlyH_101_ led to a decrease in both cell migration and proliferation, suggesting a role for functional CFTR in both processes. Remarkably, when using VRT-325 corrector to rescue F508del-CFTR traffic or transduction of CFTR cDNA into CF cells, a significant improvement in wound healing was observed [136]. Further support for a role of CFTR on cell migration emerged from a study where levels of GM1 ganglioside and sphingolipids were found to be reduced in CFTR-deficient human airway cells, thus resulting in decreased beta1-integrin signalling and delayed wound repair [137].

As in airway cells it was also shown in epithelial trophoblasts that activation of CFTR by forskolin promotes migration and its inhibition abolished the effect [138]. Furthermore, wound closure in keratinocytes from mouse epidermis lacking CFTR showed a delay compared to non-CF cells [139].

In summary, the data collected from wound healing experiments clearly show that functional CFTR promotes migration while also affecting proliferation. Thus, acting on these two processes could be a novel strategy to rescue epithelial repair in CF while contributing to promote CFTR trafficking to the PM (see Table 2). However, since such wound healing experiments were mostly performed in non-polarized cultures, it is still important to validate these results in fully differentiated polarized cells as they more closely resemble the human airway epithelium.

## 5. CFTR and Cancer

### 5.1. CF as a Disease of Increased Cancer Risk

In recent years, the median survival age for individuals with CF has dramatically increased, with adults with CF now outnumbering children in many countries worldwide. It now approaches 40 years and is predicted to reach 50 years for children born in the current era [141]. However, this long-term survival rate for individuals with CF is also recognizably associated with an elevated predisposition to cancer.

In fact, an inherent susceptibility of individuals with CF to develop cancer was described long ago. The first report from CF patients developing leukaemia and Wilms’ tumours dates from 1969 [142] and at the time occurrence of malignant tumours in patients was considered a rare event, possibly due to the fact that in the 1960’s only 20–30% of patients reached adolescence. In 1982 however, Abdul-Karim described the first pancreatic adenocarcinoma in a 27-year-old individual with CF [143], and by then researchers had started to consider that this might not be a chance association. Speculation arose that CF patients may be predisposed to this type of tumour because of the abnormal functioning of the biliary system in CF. This first account was followed by several other reports of cancer in people with CF, most being carcinomas of the digestive tract (e.g., pancreatic [144,145] and ileal [146,147] adenocarcinomas, but also leukaemia [142,148]).

However, the first substantial conclusion relating cancer to CF resulted from cohort studies performed to determine whether CF should be added to the list of conditions that increase the risk of cancer, published in 1991–1995 [1,149,150]. The 1991 Minnesota cohort, comprising 712 individuals with CF followed-up between 1962 and 1990, was unable to find evidence of an increased risk of cancer [149]. It is important to note however, that only 10% of this cohort was over 30 years. On the other hand, the 1993 UK and Wales cohort, following up 412 individuals with CF between 1961 and 1989, found a possible association between CF and adenocarcinomas of the pancreas and of the terminal ileum [150]. However, the first large international cohort of individuals with CF was the one included in the 1995 study, with 24,500 individuals with CF from Europe and 28,511 from US and Canada being monitored between 1985 and 1992. With this large number of subjects it was possible to find for the first time in individuals with CF a significantly increased risk of digestive tract cancers, namely those of oesophagus, stomach, small and large intestine, colon, liver, biliary tract, pancreas, and rectum, but not of other types of cancer [1]. By 1995, two notions were emerging: that the risk of cancer in individuals with CF might only be important if the patients survived > 25 years [149], as most of the cancers manifested in patients over 30 years [1], and that the high incidence of cancer could be caused by persistent pathological alterations inherent to CF disease in the digestive organs. 

Nonetheless, it was not until 2013 that the most comprehensive cohort study on this topic was published. This US study followed up 41,188 individuals with CF between 1990 and 2009, at a time when life expectancy was reaching 40 years. Results from this work confirmed an increased risk of digestive tract cancer (particularly following transplantation) but also an increased risk of lymphoid leukaemia and testicular cancer, and a decreased risk of melanoma (restricted to individuals homozygous for F508del) [2]. These results unequivocally proved that the risk of cancer in CF was more complex than originally thought and not just restricted to the digestive tract.

As of 2018, the CF colorectal cancer (CRC) screening task force was recommending routine screening colonoscopies for individuals with CF beginning at 40 years for non-transplanted individuals and at 30 years for those with transplants [151]. Although at present routine screening appears to be the best way to lower the risk of CRC in persons with CF, it is ultimately important to understand the multi-layered aspects of the disease in order to progressively adopt the best therapies and standards of care for individuals with CF. A recent case was reported of an individual with CF (F508del/L927P genotype) and with a primary carcinoma of the lung which metastasized to the lymph nodes, liver, adrenal glands and bone [152]. This case raises the question of whether this was a sporadic situation or lung cancer will become more common in aging people with CF and if these tumours will be more aggressive than normal. Immunotherapy in combination with chemotherapy was given to the individual in that case, which caused rapid deterioration of lung function and death, proving this to be an ineffective therapeutic route [152]. Treating cancer in subjects with CF may prove more difficult than initially thought, as it is instrumental to adequately tailor the therapies that deal with the cancer so as to avoid inflicting more damage on the CF-affected organs. A better understanding of the roles and interactors of CFTR in different organs in both health and disease may help solving this unmet need.

### 5.2. CF Carrier Status and Cancer Risk

Incidence of cancer among CF carriers is not consensual. Although some studies found that CF carriers appeared to be protected from melanoma [153,154], lung cancer [155] and prostate cancer [156], CF carriers were also reported to be associated with a modest increase in risk of pancreatic cancer, particularly those with earlier onset [157,158]. Another study reported no differences in incidence of breast cancer among F508del-CFTR carriers vs. the general population, but CF carriers presented more aggressive tumour phenotypes than non-carriers [159]. Although a significantly increased risk of kidney, thyroid, endocrine, lymphoma and non-melanoma skin cancers was found in a Swedish cohort of individuals with CF, this was not registered among their parents and siblings carrying only one mutant copy of the CFTR gene [160]. However, a very recent study assessing the prevalence of CF-related diagnostic conditions among 19,802 CF carriers, found that these individuals have a significantly increased risk of several CFTR-related conditions (e.g., pancreatitis, male infertility, bronchiectasis, diabetes) in comparison to controls, including gastrointestinal and pancreatic cancer [3]. Extra studies with similarly robust cohorts may be needed to address this question.

### 5.3. Is CFTR a Tumour Suppressor Protein?

From 1990 onwards, an increasing number of studies reported a possible role for CFTR both as an oncogene and as a tumour suppressor gene in several types of cancer. The concept that CFTR was an oncogene was first proposed by Warren [153] who postulated that the high incidence of CF carriers might derive from some biological advantage. Many other early studies supported this hypothesis, claiming an inverse association between CFTR gene mutations and the incidence of several cancers, thus suggesting that mutant CFTR (in its heterozygous form) might have a protective role against certain cancers. F508del carriers appeared to be protected from developing melanoma [153,154] and lung cancer [155]. The CFTR IVS8-5T polymorphism, associated with low CFTR expression levels, was also reported to be protective against prostate cancer in the Chinese population [156]. CFTR mutations were also reported to suppress breast cancer growth in mice by elevating extracellular ATP levels [161].

However, more recently, an increasing number of reports with better study design and including older individuals with CF started to establish a positive correlation between CF and cancer. In parallel, many studies report an actual functional role for CFTR in cancer suppression, while dysfunctional CFTR promotes cancer development both in vitro and in vivo. These studies are usually based on decreased CFTR expression levels during tumour development. Gene/promoter hypermethylation is one of the known epigenetic mechanisms by which cancer cells seem to downregulate the expression of several tumour suppressor genes. Consistently, one study reported that CFTR was found to be aberrantly methylated in 100% of the cases studied (25 out of 25) of early stage hepatocellular carcinomas (HCCs) but not in healthy liver tissue, suggesting cancer-specific hypermethylation of CFTR [162]. CFTR was also found to be one of the topmost frequently methylated genes in bladder cancer, with aberrant CFTR methylation detected in 55% (73 out of 132) of the cases [163]. Other studies found a high frequency (64%) of CFTR methylation in prostate cancers with a high malignancy score and high proliferation levels, i.e., poorer prognosis [164]. Methylation of CFTR gene was also reported to be high (30.2%) in non-small cell lung carcinoma (NSCLC) tissue samples and related to significantly lower CFTR expression in these samples in comparison to normal lung tissue [23]. More recently, promoter hypermethylation of CFTR was found in breast cancer [165]. Notably, all the patients with hypermethylated CFTR (19 out of 19) presented invasive carcinomas. CFTR CpG island hypermethylation and its consequent downregulation have also been found recently in head and neck cancer [166] and head and neck squamous cell carcinoma [167] by two independent studies. These data suggest that hypermethylation of CFTR might be a conserved mechanism for cancer cell survival across different tissues.

Other mechanisms may also be the cause of CFTR downregulation in cancer, including altered cell signalling or microenvironment changes within tumours. Whatever the cause, CFTR downregulation has been reported in cancer tissues and cells from pancreas [168], prostate [18], breast [18], lung [169,170] and respiratory tract [25], oesophagus [171], colon [24,172] and brain [173]. This downregulation was usually associated with poor prognoses [24,25,169], poor survival rates [172,173] and/or increased malignant behaviours, e.g., metastasis [25,169,170,171]. 

Downregulation of CFTR has also been linked to several known cancer pathways. For example, CFTR downregulation in pancreatic cancer cells leads to increased levels of tumour-linked mucin MUC4, altering the growth and behaviour of pancreatic adenocarcinomas [168]. Three independent studies in different tumours (prostate, breast and lung) reported very similar findings, linking the tumour-suppressing effect of CFTR to its normal role in repressing the urokinase plasminogen activator (uPA), a central protein in cell proliferation, angiogenesis, extracellular matrix degradation, invasiveness and metastasis during cancer development [18,169]. Interestingly, while in prostate cancer CFTR was reported to upregulate tumour suppressor miR-193b (which suppresses uPA) [174], in breast cancer CFTR seems to repress uPA by inhibiting NF-κB, a known uPA activator [18]. It is also possible that the two mechanisms occur in parallel, rendering CFTR a pleiotropic tumour suppressor. CFTR was also found to inhibit the growth and migration of oesophageal cancer cells by downregulating NF-kB expression [171].

Another study reported that CFTR downregulation in human CRC resulted in increased degradation and reduced stability of the AJ protein AF-6/afadin through the AF-6/MAPK pathway. The disruption of the interaction between CFTR and AF-6/afadin resulted in reduced epithelial tightness and enhanced malignancies [24]. CFTR-deficient intestinal tumours were also proposed to result from a combination of long-term chronic inflammation, microbial dysbiosis and altered innate and adaptive immune responses, with the loss of CFTR ultimately leading to β-cat activation and subsequent Wnt signalling-associated tumorigenesis [172]. CFTR activation was also reported to suppress glioblastoma cell proliferation, migration and invasion, this likely occurring through the inhibition of JAK2/STAT3 signalling [173].

Altogether, these studies suggest that CFTR interactors/pathways may be tissue/cancer specific. A full account of the association of CFTR mutations or its aberrant expression with the clinical implications in several cancer types is available elsewhere [26]. Importantly, most authors now seem to agree that apical PM expression/function of CFTR is required for the maintenance of epithelial differentiation (in terms of both polarity and tightness) and for the suppression of EMT (see below) and cancer progression/malignancy.

An exception, however, seems to be cervical/ovary cancer. Overexpression of CFTR was closely associated with cancer progression, aggressive behaviour and poor prognosis of cervical cancer [175], later associated with constitutive activation of NF-κB [176]. CFTR expression was also significantly increased in ovarian cancer where it correlated positively with the tumour progression state and malignancy degree (cell invasion, motility and proliferation) [177]. Although further studies are needed to better understand this phenomenon, a possible explanation is that oestrogen, frequently abnormally increased in ovarian and cervical cancers, is known to stimulate CFTR expression, which could happen in this case [26,178]. This, however, would be a secondary occurrence and not a causal one.

An interesting study found that F508del-homozygous human epithelial foetal tracheal cells display a pro-angiogenic state compared to controls. The media in which these CF cells were cultured induced proliferation, migration and sprouting of cultured primary endothelial cells, thus suggesting a possible link between proliferation/ invasiveness and absence of functional CFTR [179].

Although many authors have reported that downregulation of CFTR leads to tumorigenesis and/or invasiveness it is still largely unknown how this occurs. Thus, further studies are needed in order to better understand the elusive moonlight role(s) of CFTR in cancer. Some light on the role of CFTR in cancer seems to be shed by its connection to the process of epithelial–mesenchymal transition (EMT) which is discussed in the next section.

## 6. CFTR and Epithelial–Mesenchymal Transition (EMT)

**Figure 4:**
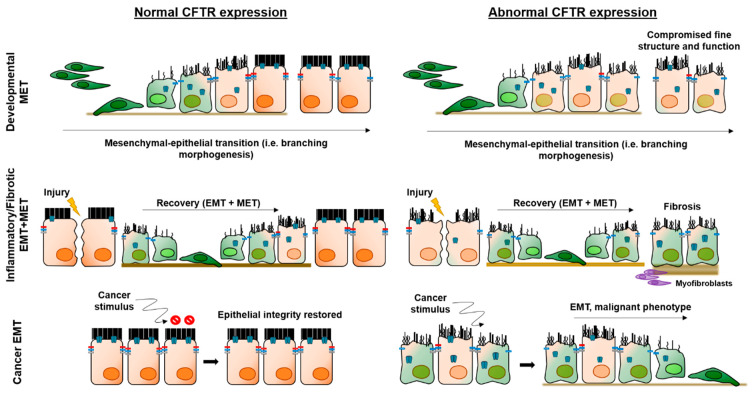
Proposed roles of CFTR in the three different epithelial–mesenchymal transition (EMT) settings.

EMT is a latent, developmental process, which involves transcriptional reprogramming of epithelial (differentiated) cells into mesenchymal (primordial) cells (Figure 4). The EMT programme is driven by EMT-associated transcription factors (EMTa-TF) which inactivate genes encoding epithelial-specific proteins while overexpressing genes that define the mesenchymal phenotype. This process results in the loss of apical-basal polarity and of cellular junctions, changes in cell shape and enhanced migratory properties [180,181]. Depending on the biological setting, EMTs can be grouped into three types. Type 1 is developmental EMT which is critical for tissue morphogenesis and organogenesis and is silent in healthy adult tissues. Types 2 and 3 represent pathological conditions in which the EMT programme is reactivated in inflammatory diseases (Type 2) or cancer (Type 3) [182].

Because of the emerging roles of CFTR as a tumour suppressor and as a key player in development and epithelial differentiation, it is plausible to envisage its involvement in EMT. The rationale is simple: CFTR would be required for mesenchymal–epithelial transition (MET) during development and afterwards to maintain the differentiation state of epithelial cells functioning as a protector from EMT (Figure 4).

CFTR downregulation due to TGF-β1-treatment of differentiated primary human bronchial epithelial cells was first reported in 2013, along with an increase in levels of N-cadherin, but no decrease of E-cadherin [183]. Since a switch from E- to N-cadherin is the hallmark of EMT, the authors ruled out EMT as no E-cadherin decrease occurred in these cultures. However, this notion has more recently been challenged, as partial EMT is now generally accepted as a spectrum of states in which cells display intermediate epithelial/mesenchymal phenotypes (Figure 4) [184]. In fact, a recent transcriptome profiling meta-analysis revealed an EMT signature in the airways of individuals with CF [19]. Moreover, it was also recently shown that partial EMT is intrinsically triggered by the absence of functional CFTR, thus suggesting that CFTR plays a direct role in EMT protection (M Quaresma and Amaral lab, unpublished data). In this partial EMT state, CF tissues/cells display destructured epithelial protein distribution, expression both of mesenchymal markers and of EMTa-TFs, besides hyper-proliferation, impaired wound-healing and over-sensitivity to TGF-β induced EMT. Interestingly, this CF EMT phenotype could be partially reverted by drugs that rescue CFTR. A similar protective role for CFTR in EMT induction was also found recently by other authors, reporting that CFTR activity led to increased cellular tension across E-cadherin, thus preventing expression of mesenchymal markers and EMTa-TFs [22].

Two other studies have suggested that CF modifier genes (but not CFTR directly) FAM13A and tissue transglutaminase 2 (TG2) are involved in EMT. FAM13A is a small GTPase that, when downregulated, was implicated in idiopathic pulmonary fibrosis (IPF) [185] and surprisingly, when overexpressed, was implicated in other respiratory diseases, like chronic obstructive pulmonary disease (COPD) or cancer [186]. When downregulated by TGF-β1, FAM13A is responsible for EMT progression, something that likely also happens in CF lung disease [187]. As for TG2, it was found to be elevated in CF epithelial cell cultures and shown to decrease CFTR stability, activate TGF-β1 and induce fibrotic EMT [20]. EMT is a known driver of tissue remodelling and fibrosis in inflammatory diseases, including COPD and IPF [188] and CF shares both clinical features (obstructed airways, chronic inflammation and fibrosis) and gene expression profiles with those conditions [19]. An important driver of EMT is TGF-β1, which is overexpressed in CF, as well as in those chronic airway diseases [189]. A possible involvement of CFTR in partial (Type 2) EMT has been discussed elsewhere [21]. Notwithstanding, despite appearing as a plausible contributor to CF disease (due to its fibrotic nature), there are so far very few direct reports linking CFTR to this process. One study has indeed connected downregulation of CFTR in renal epithelial cells to dysregulated Wnt/β-cat signalling leading to activation of EMT and kidney fibrosis [190].

More concrete evidence for a role of CFTR in more advanced EMT (Type 3) has been established by studies on the role of CFTR as a tumour suppressor, some authors suggesting a direct (causal) link between the downregulation of CFTR in tumour cells and EMT induction. This is the case for breast cancer, where the EMT-suppressing effect of CFTR (i.e., increase in the levels of E-cadherin and decrease in the levels of vimentin) was associated with its ability to inhibit both NF-κB and uPA [18]. Similar findings were produced in NSCLC, where CFTR inhibition activated the uPA/uPAR pathway and generated changes in cell morphology, downregulation of E-cadherin and upregulation of vimentin and fibronectin [169]. Additionally, in CRC, inhibition of CFTR was found to downregulate epithelial markers and upregulate mesenchymal markers, consistent with an EMT signature [24]. More recently, mouse F508del-CFTR embryonic stem cells were found to form more aggressive teratomas than wt-CFTR, with enhanced cell proliferation, migration and upregulation of EMT-associated genes, including, once again, uPA [191].

Hence, although further studies need to be conducted to better elucidate the mechanisms involved, it appears that CFTR plays a role in the EMT process.

## 7. Conclusions

An increasing number of emerging studies show that CFTR plays a role in fundamental cellular processes which include development, epithelial differentiation/polarization, regeneration, migration and proliferation as well as in EMT and cancer. Whether the disruption of these processes is a direct or indirect consequence of dysfunctional CFTR remains to be elucidated. Moreover, if indeed these processes directly require functional CFTR, the compelling question is whether they are related to the anion channel role of CFTR or to another, yet to be identified, “moonlighting” function of CFTR.

## Figures and Tables

**Table 1:** Levels of CFTR mRNA expression during human foetal development.

Tissues	1st Trimester	2nd Trimester	3rd Trimester	References
Pancreas	+++	+++	+++	[27,30,31,32]
Liver	++	++	++	[27,31]
Kidneys	++	+++	++	[33]
Colon and small intestine	+++	+++	+++	[10,27,31]
Large airways	+ d	+	+	[27,28,29,31]
Small airways	+	++	+	[27,28,31]
Submucosal glands	ND	ND	ND	[28]
Epididymis Vas deferens	+	+	+	[30,31]
Uterus Fallopian tubes	ND	ND	+	[31]

Legend: +++ high, ++ medium, + detectable, d diffuse, ND not detectable.

**Table 2:** Differential expression of proteins/markers in cystic fibrosis (CF).

Protein/ Marker	Reported Difference in CF	Cells/Tissue	Assay	Reference
Actin stress fibers	Disorganized	CF (CFBE41o-) vs. non-CF (16HBE14o-) cells	Immunostaining	[103,104]
Actin stress fibers	Not present in CFBE41o- compared to 16HBE14o- Some present in CFBE41o-/wt-CFTR	CF (CFBE41o-) vs. 1 non-CF (6HBE14o-) vs. isogenic non-CF (CFBE41o-/wt-CFTR) cells	Immunostaining	[105]
KRT5 KRT14 EGFR	Increased	Airway sections from human CF and non-CF explanted lungs	Immunohisto-chemistry	[126]
ZO-1	Increased when cells cultured at 29 °C compared to 37 °C	CF (CFBE41o-) cells	Immunostaining	[14]
ZO-1 Occludin Cldn1 JAM-1	Not expressed	CF (CFBE41o-) vs. non-CF (16HBE14o-) cells	Immunostaining	[110]
Cldn1 Cldn7 Cldn8 Pmp22	Decreased	CF and non-CF mouse intestine	qRT-PCR Immunohisto-chemistry	[116]
Cldn2	Increased	CF and non-CF mouse intestine	qRT-PCR Western Blot Immunohisto-chemistry	[116]
Cldn3	Decreased in CFBE41o-, wt-CFTR, F508del-CFTR compared to 16HBE	CF (CFBE41o-), isogenic non-CF (CFBE wt-CFTR) or CF (CFBE F508del-CFTR) vs. non-CF (16HBE14o-) cells	Immunostaining	[140]
Connexin-43	Mislocalized	CF (CuFi-5) vs. non-CF (NuLi-1) cells	Immunostaining	[113]
TEER	Decreased in GFP-F508del-CFTR expressing cells vs. GFP-wt-CFTR	CF (CFBE41o-) cells	Volt-Ohm Meter	[14]
TEER	Decreased	CF (CFBE41o- vs. non-CF (16HBE14o-) cells	Volt-Ohm Meter	[112]
TEER	Lower	CF (CuFi-5) vs. non-CF (NuLi-1) cells	Volt-Ohm Meter	[113]
Epithelium height	Increased	CF and control human airway tissue	Histological examination	[16]
Ki-67 KRT13	Increased	CF and control human airway tissue	Histological examination	[16]
MUC5B	Reduced	CF and control human airway tissue	Histological examination	[16]
IL-8 MMP-7 MMP-9 TIMP-1	Increased	CF and control human airway tissue	RT-qPCR	[16]
PCNA	Increased	CF and control human airway tissue	Immunostaining	[125]
Ki-67	Increased	CF and control lung sections	Immunohisto-chemistry	[126]
KLF4 KLF2	No change during repair of CF HAEC	CF HAEC and non-CF HAEC	RT-qPCR	[127]
Ki-67 Connexin-26	Elevated in CF HAEC during repair	CF HAEC and non-CF HAEC	Immunostaining	[127]
Cilia biology related genes	Downregulated	CF Nasal Epithelia	Microarray	[95]
Proliferation related genes	Upregulated	CF and non-CF Nasal Epithelia	Microarray	[95]
β-Tubulin	Lower	CF and non-CF human airway epithelial cells	Immunostaining	[128]

## Abbreviations

AJ: adherens junction(s); ALI, air-liquid interface; β-cat, β-catenin; CBAVD, congenital bilateral absence of the vas deferens; CF, Cystic Fibrosis; CFTR, CF transmembrane conductance regulator; CK, cytokeratin; CNX43, connexin-43; COPD, chronic obstructive pulmonary disease; CRC, colorectal cancer; DP, desmoplakin; EMT, epithelial–mesenchymal transition; EMTa-TF, EMT-associated transcription factor; FGF, fibroblast growth factor; GJ, gap junction(s); IPF, idiopathic pulmonary fibrosis; mESC, (mouse) embryonic stem cell(s); MET, mesenchymal–epithelial transition; NHERF1, Na^+^/H^+^ exchanger regulatory factor isoform 1; NSCLC, non-small cell lung carcinoma; PGC, primordial germ cell(s); PKA, protein kinase A; PM, plasma membrane; RARα, retinoic acid receptor alpha; ROCK, Rho-associated protein kinase; sAC, soluble adenylate cyclase; SLC26A3/ SLC26A6, Solute Carrier Family 26 Member 3/6; TEER, transepithelial electrical resistance; TGFβ1, transforming growth factor β1; TJ, tight junction(s); uPA, urokinase plasminogen activator; VIP, vasoactive intestinal peptide; WG, weeks of gestation, ZO-1, zona occludens (protein) 1.
